# Rope on Rope: Reducing Residual Vibrations in Rope-Based Anchoring System and Rope-Driven Façade Operation Robot

**DOI:** 10.3390/s25082463

**Published:** 2025-04-14

**Authors:** Kangyub Lee, Sahoon Ahn, Jeongmo Yang, Hwasoo Kim, Taewon Seo

**Affiliations:** 1 Mechanical Engineering, Hanyang University, 222, Wangsimni-ro, Seoul 04763, Republic of Korea; skymusic7@hanyang.ac.kr (K.L.); noohasnha@hanyang.ac.kr (S.A.); didwjdah5959@hanyang.ac.kr (J.Y.); 2 Department of Mechanical System Design, Kyonggi University, Suwon 16227, Republic of Korea; hskim94@kgu.ac.kr

**Keywords:** rope riding, rope-on-rope model, cable-driven parallel robots, input shaping, disturbance observer

## Abstract

Maintenance of the exteriors of buildings with convex façades, such as skyscrapers, is in high demand in urban centers. However, manual maintenance is inherently dangerous due to the possibility of accidental falls. Therefore, research has been conducted on cleaning robots as a replacement for human workers, e.g., the dual ascension robot (DAR), which is an underactuated rope-driven robot, and the rope-riding mobile anchor (RMA), which is a rope-riding robot. These robots are equipped with a convex-façade-cleaning system. The DAR and RMA are connected to each other by a rope that enables vibration transmission between them. It also increases the instability of the residual vibration that occurs during the operation of the DAR. This study focused on reducing the residual vibrations of a DAR to improve the stability of the overall system. Because it is a rope-on-rope (ROR) system, we assumed it to be a simplified serial spring–damper system and analyzed its kinematics and dynamics. An input-shaping technique was applied to control the residual vibrations in the DAR. We also applied a disturbance observer to mitigate factors contributing to the system uncertainty, such as rope deformation, slip, and external forces. We experimentally validated the system and assessed the effectiveness of the control method, which consisted of the input shaper and disturbance observer. Consequently, the residual vibrations were reduced.

## 1. Introduction

Cities are progressing due to rapid industrial expansion. As the role of cities increases, the number of buildings that house the population also increases. Advancements in construction technology have made it possible to build tall buildings. Meanwhile, the number of buildings with atypical shapes that fulfill human aesthetic needs, such as six-sided skyscrapers, has increased. The most common method of cleaning high-rise exterior walls is to do it manually. However, the height and convex shape of these buildings increase the difficulty of cleaning the exterior walls and create a hazardous working environment for workers.

**Figure 1:**
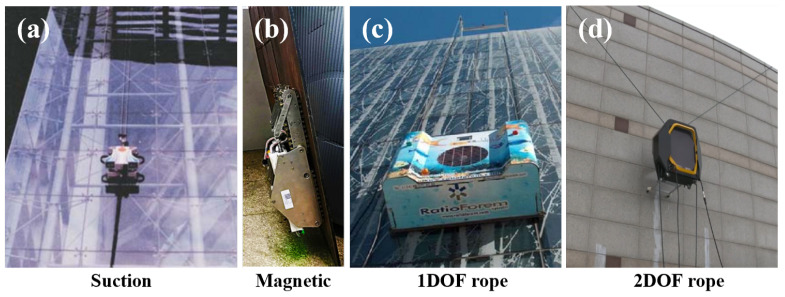
Classification of robot types: (**a**) suction-based [1], (**b**) magnetic-based [6], (**c**) rope-driven with 1 degree of freedom (DOF) [10], and with (**d**) 2 degrees of freedom (DOF) [11].

As shown in Figure 1, various forms of high-rise cleaning robots and exterior climbing mechanisms have been developed to reduce the cost of cleaning and risk to workers. The Sky cleaner series [1,2] and SkyScraper I [3] are examples of robots that climb exterior walls using suction cups that generate a vacuum. Suction cup-type robots easily move on the façade. However, they can only be used on smooth surfaces such as glass but not on convex façades or rough surfaces such as metals. Robots that use magnetism to climb exterior walls include mobile robots with magnetic wheels [4,5], with magnetic treads [6,7], and with magnetic caterpillar tracks [8,9]. Robots using ropes or lines to climb walls have also been developed.

As the shapes of buildings become more diverse, it is necessary to develop a cleaning robot for convex façades in addition to traditional flat exterior walls. A robot that climbs façades using a rope was evaluated as suitable for developing a façade-cleaning robot that can be applied to all types of buildings [10,12]. However, they can only move vertically, which has a disadvantage in that the line installation location must be moved after each step to clean the entire wall. It also requires the installation of additional complex external infrastructure. To overcome this limitation, Edelsro-M2 [11], which can move with two degrees of freedom, was developed. It utilizes a dual rope-ascension mechanism and parallel kinematics by connecting the ropes to two anchors fixed at the top of the building. This method eliminates the need for additional infrastructure and allows them to adapt to any exterior wall using a pair of ropes. This mechanism increases the work space of the robot along the length of the rope because the other end of the rope is not fixed. It also allows for smaller and lighter robots because the entire rope is not wrapped inside the robot. The ascension mechanism simplifies the control of the robot in large spaces, because the weight of the elevation mechanism does not change as the robot moves due to the constancy of the total rope length.

**Figure 2:**
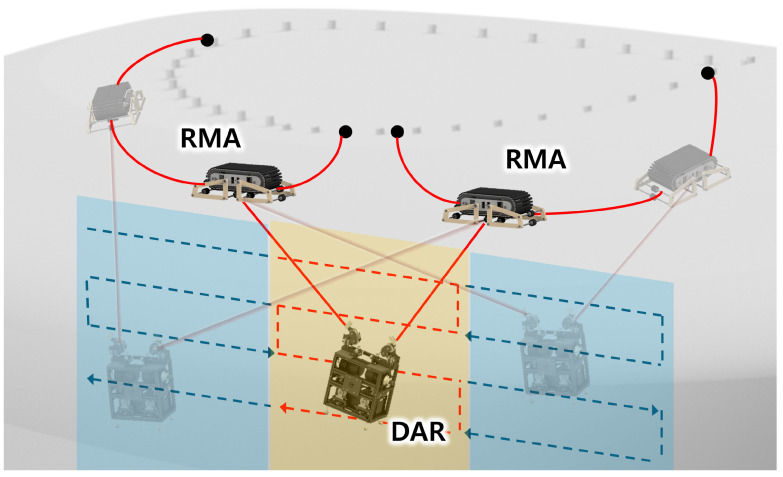
Concept of the rope-on-rope (ROR) system: the dual ascension robot (DAR)’s rope is connected to a rope-riding mobile anchor (RMA). The yellow area indicates the original DAR workspace, while the blue area represents the expanded DAR workspace enabled by the movement of the RMA.

In addition, a fixed position of the anchors implies that they are only accessible within the anchor area, and the tension constraints of the ropes result in areas of low manipulability, which refers to the ability to reach a specific location or set of locations [13]. To address the limited access problem, M-CDPR has been studied for CDPR, which can overcome the interference problem in a limited workspace by moving the anchor points [14,15,16]. As shown in Figure 2, a rope-riding mobile anchor (RMA) robot was developed [17] for a DAR, which expands the cleaning area of a façade-cleaning robot for both flat and convex façades. It also enhances the manipulability problem caused by rope tension by moving the anchor point. The rope-riding mechanism has several advantages. For example, it does not require a pre-installed winch on the exterior wall, and the rope is easy to install and remove, which enables convenient workspace expansion. However, this mechanism transmits additional rope vibrations to the DAR connected to the rope, thereby causing instability.

This study aims to enhance the stability of the system by reducing residual vibrations during DAR operation. Because the RMA is a robot riding on a fiber rope and the DAR is coupled with it, the system can be considered a rope-on-rope (ROR) system. Since previous RMA research [17] did not consider the vibrations of the ROR system, we analyzed its kinematics and dynamics. In this process, we modeled the rope as a spring–damper system, as several previous studies have adopted this approach [18,19,20]. In particular, ref. [21] defined the deformation length of the elastic rope and designed a feed-forward controller to predict the tension and slippage caused by rope elasticity, thereby reducing positional errors.

However, the characteristics of the rope aggravate the vibrations, and it is necessary to attenuate them. As mentioned in [17], the ROR system has a limitation in that the two robots cannot move simultaneously, making it difficult to utilize the RMA for vibration reduction during the DAR’s motion. To ensure stable movement of the DAR, the RMA employs a fixed mechanism that firmly anchors itself to the rope, and when the RMA needs to move, this mechanism is temporarily released. If the DAR moves during this period, the resulting oscillations may cause the RMA to slip, compromising its stability. Studies [22,23] have explored vibration reduction by integrating additional hardware components such as reaction wheels or thrusters. Nevertheless, such approaches face limitations, including increased system weight, noise generation, and a decrease in operational speed. While control algorithms like Nonlinear Model Predictive Control (NMPC) have shown promise in dynamic environments [24], their direct application to our ROR system faces limitations. NMPC relies on precise system models as shown in hierarchical control for mobile robots [25,26], but it conflicts with the inherent uncertainties in fiber ropes such as deformation and slipping. Similarly, RL-based approaches like DDPG require extensive training data that are impractical to collect safely in high-altitude building environments [27].

Given these constraints, we prioritized a hybrid approach combining input shaping and disturbance observers (DOBs). Input shaping is a feed-forward control technique used to reduce residual vibrations in flexible or underdamped systems by convolving the desired input with a sequence of impulses. As shown in [28,29], input-shaping techniques have been successfully applied to suppress residual vibrations in overhead crane systems, which exhibit motion characteristics similar to those of the DAR. Also, refs. [22,30] constrained vibrations along the X- and Y-axes; however, this could not be achieved along the yaw direction. And a DOB is a control algorithm that estimates and compensates for external disturbances or model uncertainties. Since we experienced issues with rope deformation and slipping in a previous study [21], which contributed to uncertainties in system control, a DOB was used to inspect and remove these disturbances. Finally, we created and experimentally validated a simplified alternative robot prototype of a DAR.

The remainder of this article is organized as follows. In Section 2, a mathematical analysis of the ROR system, comprising the DAR and RMA, is conducted to explore its dynamic characteristics. In Section 3, based on the dynamic analysis and experimental data, the disturbance observer and input shaper are designed to effectively suppress the residual vibrations. In Section 4, the reduction of residual vibrations in the ROR system is experimentally verified. Finally, the conclusions of the study are provided in Section 5.

## 2. Rope-on-Rope (ROR) System Modeling

### 2.1. System Configuration

Figure 2 illustrates the ROR system. The DAR’s overall design comprises two independently operated rope winches, a rope guide capable of passive movement in response to the direction of rope tension, and an angle-measurement system that determines the robot’s position and orientation by calculating the angle of the rope guide. Each rope winch was powered by an independent motor, which allowed the rope length to be adjusted by rotating the traction pulley in either direction. If the end of the rope in the DAR was not secured, the rope might lose its tension, causing the DAR to fall. To prevent slippage and maximize friction between the traction pulley and the rope, a tension-maintaining bearing was installed at the rope end.

The RMA is a rope-riding robot that moves on a rope while carrying a heavy payload, i.e., the DAR. As the RMA remains non-driving during the movement of the DAR, as described in [17], it is assumed to be a simple mass without active movement, replacing it with a mass. The mathematical symbols used in the model are listed in Table 1.

### 2.2. Kinematic Analysis

**Figure 3:**
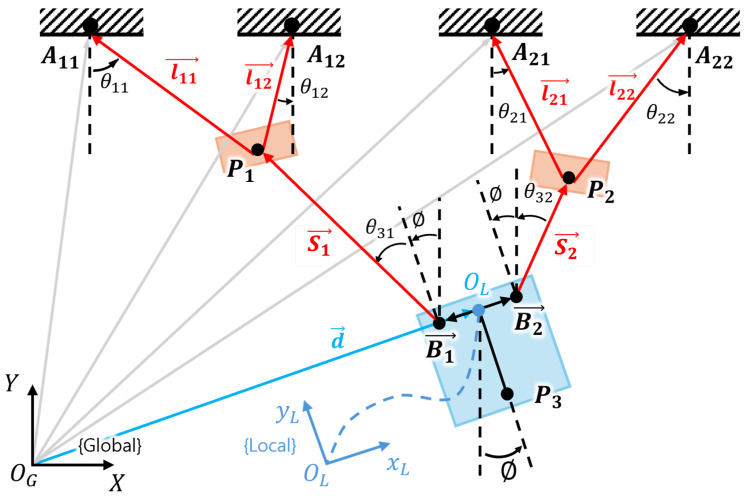
Kinematic model of the ROR system: $\theta_{i}$ denotes the rope’s angle. Red lines denote the vectors of the ropes. The gray lines denote the vectors of the fixed anchors, and the blue line denotes the vector of the DAR’s local coordinate system. The red boxes represent the RMA and the blue box the DAR. $P_{1}$ and $P_{2}$ are the positions of the center of mass of the RMAs, and $P_{3}$ represents the center of mass of the DAR. Local coordinates are defined with the origin at the midpoint $O_{L}$, between two magnetic encoders.

The kinematic model of the ROR system is expressed as shown in Figure 3, where the kinematic constraints on the ROR system can be derived as follows:

(1) $$\vec{A_{ij}}=\vec{d}+\vec{B_{i}}+\vec{S_{i}}+\vec{l_{ij}},\;i,j=1,2$$

where $\vec{A_{ij}}$ is the vector from the origin of the global coordinate system to the fixed anchor, $\vec{d}$ is the vector from the origin of the global coordinate system to the origin of the local coordinate system of the DAR, $\vec{B_{i}}$ is the vector from the origin of the local coordinate system of the DAR to each magnetic encoder, and $\vec{S_{i}}$ and $\vec{l_{ij}}$ denote the vectors of the ropes. Using forward kinematics, the RMA and DAR’s coordinates (X), Jacobian matrix ($J_{1}$), and lengths of rope (**q**) are expressed as follows:

(2) $$\dot{X}=J_{1}^{T}\dot{q}$$

where **X** = ${\left[x_{1},y_{1},x_{2},y_{2},x_{3},y_{3}\right]}^{T}$ and **q** = ${\left[l_{11},l_{12},l_{21},l_{22},S_{1},S_{2}\right]}^{T}$.

### 2.3. Dynamic Analysis

**Figure 4:**
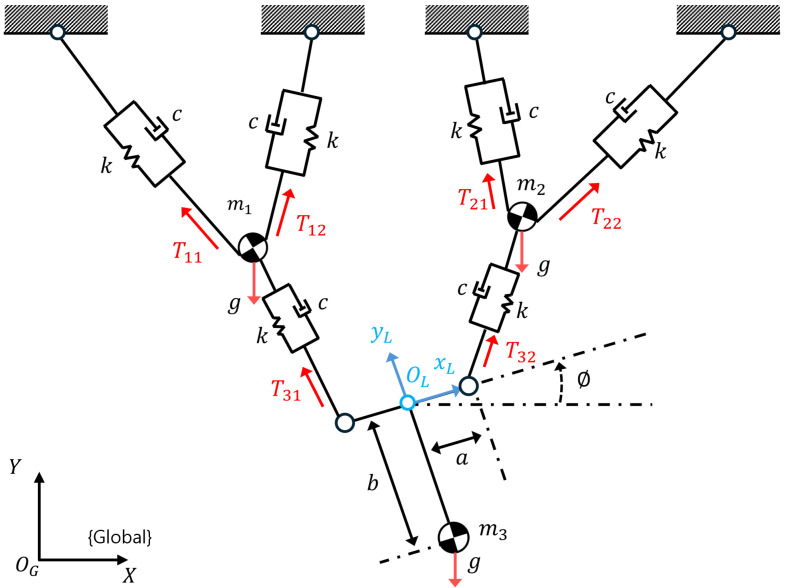
Simplified dynamic model of the yaw motion in an ROR system: for simplicity of modeling, the entire mass of the robot is assumed to be at the center of gravity. In the figure, $m_{1}$ and $m_{2}$ denote the masses of RMAs, while $m_{3}$ denotes the mass of the DAR.

A dynamic model of the ROR system consisting of RMA and DAR is shown in Figure 4, and the equations of linear and rotational motions of the system are derived as follows:

(3) $$M\ddot{X}+J_{2}T=G$$

(4) $$I\ddot{\phi }+J_{3}T=0$$

where **M** denotes the robot’s mass matrix, X denotes the coordinates of the RMAs and the DAR, and I denotes the moment of inertia of the robot. $J_{2}$ and $J_{3}$ denote dynamic Jacobian matrices. **T** denotes the rope tension due to strain, and **G** denotes the gravitational force on the robots, which is the only external force on the system. Modeling a rope is difficult because it contains several threads braided into a spiral. To express the tension in the rope, we modeled the rope as a simplified spring–damper system [30]; therefore, **T** can be represented as follows:

(5) $$T=KU\left(q,u_{i}\right)+CU\left(\dot{q},\dot{u_{i}}\right)$$

where **K** denotes the stiffness of the rope tension, **C** denotes the damper coefficient matrix, and $U(q,u_{i})$ denotes the change in the rope length based on the robot’s position and system input. Assuming the use of the same type of rope, we define **K** and **C** as identity matrices k and c, respectively. As in a previous study [30], k and c are determined experimentally. Because the length and cross-sectional area change as the rope is loaded, *k* is defined as follows:

(6) $$k=\frac{AE}{l_{0}}$$

where *A*, *E*, and $l_{0}$ denote the cross-sectional area, Young’s modulus, and initial length of the rope without a load, respectively. The following section describes a residual vibration control algorithm that combines input shaping with a disturbance observer.

## 3. Residual Vibration Control

Owing to the nature of the ROR system, vibrations occur during and after operation, affecting stable movement performance. This was attributed to the deformation and slippage of the rope carrying the DAR. Several studies have proposed relevant vibration control methods; however, residual vibrations in the form of rotational vibrations could not be controlled effectively [22,30]. This is attributed to the underactuated characteristics of the DAR, which can control translational movements but not rotation.

**Figure 5:**
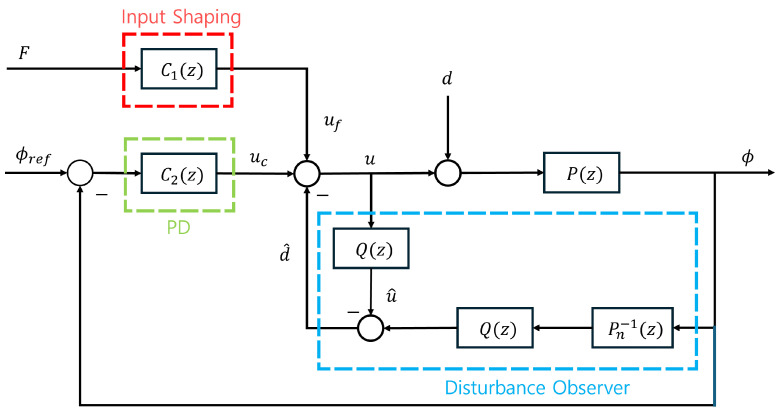
Block diagram of the ROR system’s residual vibration control algorithm (RVCA) consisting of input shaping and a distance observer; P(z) is the ROR system’s transfer function, and $P_{n}^{-1}\left(z\right)$ is its nominal plant.

This study programs a residual vibration control algorithm (RVCA), as depicted in Figure 5. This algorithm, which combines feedback and feed-forward control, can effectively attenuate the residual vibrations. Horizontal acceleration was set as the input, which led to the movement of the DAR and affected the residual vibrations. The algorithm is detailed in subsequent sections.

### 3.1. System Analysis

Considering the uncertainty and disturbance caused by the ROR structure, the transfer function between the acceleration and the rotation angle of the DAR was experimentally obtained using MATLAB R2024b’s System Identification Toolbox. Owing to the nature of the ROR system, as the DAR approaches the edge of the workspace, the tension is concentrated on one rope, and the mobility in the direction perpendicular to that tension is reduced. Therefore, the positions of the RMAs were adjusted to expand the workspace, and for stable operation, the DAR was primarily operated between the two RMAs. Phi(ϕ) was horizontal at the midline and varied insignificantly near it. Therefore, we chose the reference posture as the central position in the following experiments to obtain a linearized transfer function expression.

**Figure 6:**
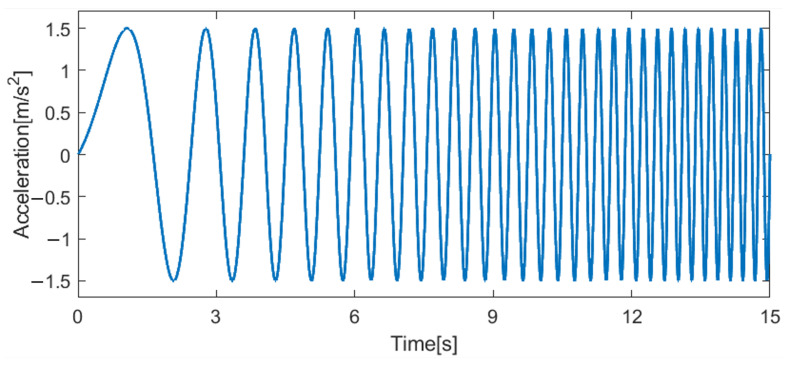
Experimental input: x-direction acceleration of DAR applied with chirping frequency of up to 2 Hz.

Experiments were conducted to collect the input–output data. The DAR’s x-axis acceleration, $\ddot{x}$, which represents the force derived by the motors that move the DAR on the x-axis, was set as the input. This mainly affects the DAR’s rotation angle, ϕ, which is the output representing the residual vibrations. As shown in Figure 6, a chirp input of x-axis acceleration up to 2 Hz was applied, and the resulting ϕ was collected. These input–output data serve as the basis for system identification.

Considering the dimensions of acceleration and angle, the degrees of the denominator and numerator differed by 2. This characteristic was incorporated into MATLAB R2024b’s System Identification Toolbox. The discrete transfer function of the ROR system was derived based on these results, as shown in Equation (7). From this equation, the natural frequency and damping ratio of the ROR system, which are essential parameters for input shaping a feed-forward control technique, were determined.

(7) $$G\left(z\right)=\frac{\phi (z)}{\ddot{x}\left(z\right)}=\frac{0.0396z^{-1}+0.0398z^{-2}}{1-2.11z^{-1}+1.136z^{-2}+0.0626z^{-3}-0.0875z^{-4}}$$

### 3.2. Disturbance Observer

The disturbance observer is a robust control method prevalent in mechatronics. It is highly effective at rejecting disturbances and compensating for plant uncertainties. Owing to the characteristics of the ROR system, the rope slips from the rope-holding pulley and deforms, which prevents the input from being fully transmitted to the system. The disturbance observer was implemented to address this issue by defining the disturbances and eliminating their impact.

In Figure 5, P(z) and $P_{n}^{-1}\left(z\right)$ are the plant Equation (7) and nominal plant of the ROR system, respectively. In this method, a low-pass filter was used as the Q-filter Q(z), which was required to implement the inverse of the nominal transfer function [31]. As Equation (7)’s order and relative order are 4 and 1, respectively, to implement $Q\left(z\right)P_{n}^{-1}\left(z\right)$, the order and relative order of Q-filter were each set to 1, as follows:

(8) $$Q\left(z\right)=\frac{\omega }{z+\omega }$$

where ω = 60, which is the cut-off frequency of the Q-filter.

### 3.3. Input Shaping

**Figure 7:**
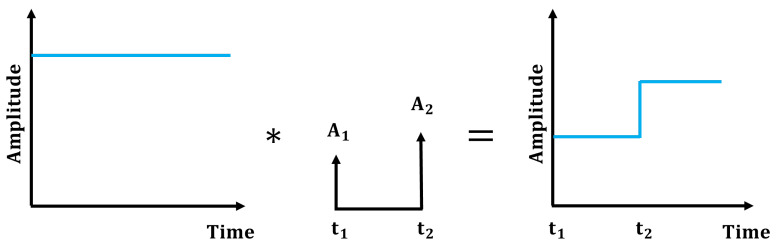
Basic concept of input shaping; it converts a sequence of impulses into the desired command via convolution represented by *, with reference inputs; $A_{1}$ and $A_{2}$.

Input shaping is an open loop compensator that modifies the actuator input to eliminate vibrational motion after the input ceases. This method is based on the concept that the superimposed impulse responses can cancel each other after the final impulse. Implementing input-shaping control involves convolving a sequence of impulses with reference inputs. The critical parameters of this scheme are the amplitudes $A_{i}$ and specified application times $t_{i}$ of the impulses, as shown in Figure 7. The system’s natural frequency $\omega_{n}$ and damping ratio ζ, which are essential for this control technique, can be derived from Equation (7); $\omega_{n}$ is 7.42 rad/s and ζ is 0.0152.

In this study, the zero-vibration shaper, which was the first input shaper designed to be robust against modeling errors [32], was implemented to reduce residual vibrations in the ROR system, detailed as follows:

(9) $$\begin{array}{c} \left[\begin{array}{c} A_{i}\\ t_{i} \end{array}\right]=\left[\begin{array}{cc} \frac{1}{1+K^{2}} & \frac{K}{1+K^{2}}\\ 0 & 0.5T_{d} \end{array}\right]\\ \mathrm{where}\;K=e^{\frac{-\zeta \pi }{\sqrt{1-\zeta }}},\;\frac{T_{d}}{2}=\frac{\pi }{w_{d}} \end{array}$$

By applying the previously derived natural frequency and damping ratio values, Equation (9) can be expressed as follows:

(10) $$\begin{array}{cc} \left[\begin{array}{c} A_{i}\\ t_{i} \end{array}\right] & =\left[\begin{array}{cc} 0.5119 & 0.4881\\ 0 & 0.4234 \end{array}\right] \end{array}$$

An acceleration trajectory was created according to Equation (10). Based on this trajectory, a position trajectory was generated as an input to the motor.

### 3.4. Control Algorithm

As illustrated in Figure 5, the RVCA combines the feed-forward and feedback control mechanisms. The input-shaping method was applied in the ROR system to minimize residual vibrations during and after the DAR operation. However, this would not eliminate the residual vibrations [30]. Because the ROR system operates in high-rise environments, it is frequently subjected to external forces such as wind.If the system encounters these external forces immediately after movement or while non-driving, the proportional derivative (PD) control should be employed to mitigate the resulting vibrations. In addition, it can reduce residual vibrations after DAR operation. We reduced the residual vibrations faster by applying the PD control after movement with the input shaping. Throughout the feedback control process, disturbance observers were employed to prevent residual vibrations from being exacerbated by disturbance-containing inputs. The next section presents a performance evaluation of the proposed control algorithm through a comparison between experiments conducted with and without the algorithm applied.

## 4. Verification Experiment (See Supplementary Materials)

In this section, we compare the residual vibrations of the DAR with and without the control algorithm, as shown in Figure 5. In addition, the simulation results obtained by modeling Equation (7) were compared with the experimental results to verify the model.

### 4.1. Experiment Settings

**Figure 8:**
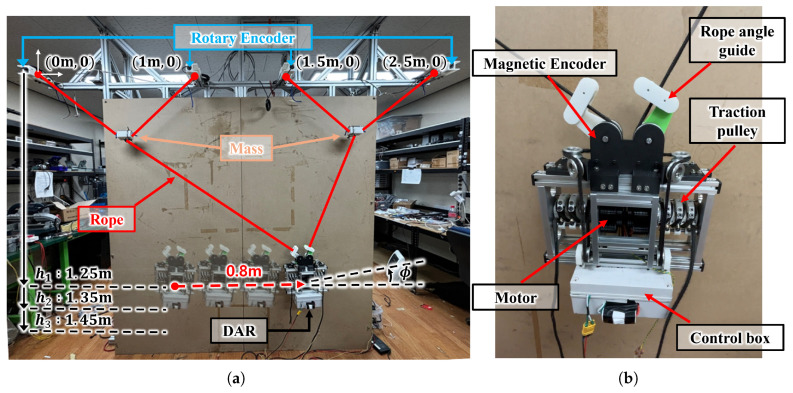
Experiment setup for verification experiment. (**a**) Test bench for experiment; rotary encoders, represented by blue lines, measure the angle of the rope to which the masses are attached, and the masses represented by orange lines replace the non-driving RMAs on the rope (red lines). The experiments are conducted at vertical distances of $h_{1}$ = 1.25 m, $h_{2}$ = 1.35 m, and $h_{3}$ = 1.45 m from the top fixed anchor point. (**b**) Simplified DAR; magnetic encoders measure the angle of the rope to which the DAR is attached, and the motor rotates the traction pulley to control the length of the rope that is fixed to it.

As depicted in Figure 8, we constructed a test bench implementing the ROR system with the simplified DAR for the experiments. Acceleration trajectories with PD control, input shaping, RVCA, and an open loop were generated for the following experiments with the same average velocity. As depicted in Figure 8a, at heights of $h_{1}$, $h_{2}$, and $h_{3}$, the DAR moves 0.8 m horizontally along the position trajectories derived from this acceleration trajectory, and phi(ϕ) is measured by IMU(E2BOX, Hanam, Republic of Korea). Rotary encoders (Autonics, Yangsan, Republic of Korea) and magnetic encoders (SEINFLEX, Anyang, Repulic of Korea) were used to calculate and adjust the position of the DAR. As described in [17], when the DAR is moving, the RMA remains non-driving. Therefore, in this experiment, the RMA was modeled as a 1 kg mass fixed to the rope. The DAR weighed approximately 6.5 kg and employed two BLDC motors (Cubemars, Nanchang, China) that wound the ropes and moved the robot. The motor inputs were determined using inverse kinematics, as defined in Equation (2). The sensors and motors used in the robot are listed in Table 2.

### 4.2. Experiment Results and Discussion

**Figure 9:**
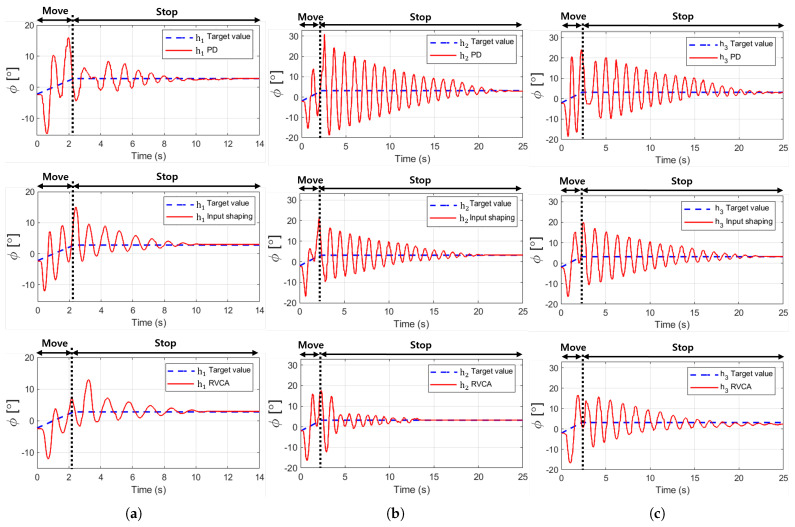
Comparison of the amount of residual vibrations expressed in ϕ. The blue lines denote the target value for that angle, and the red lines denote the result for each experimental condition; PD control, input shaping, and RVCA at heights of (**a**) $h_{1}$, (**b**) $h_{2}$, (**c**) $h_{3}$, respectively.

By conducting experiments under each condition and measuring the resulting residual vibrations ϕ, the results shown in Figure 9 were obtained. The results indicate that the RVCA most effectively reduces the residual vibration in terms of the maximum amplitude ($\phi_{max}$) and settling time ($T_{s}$). Owing to the kinematic features of the ROR system, as shown in Figure 3, the local coordinate of the DAR rotates in the negative direction if the DAR is located on the left of the center, and in the positive direction if it is located on the right. As the local coordinates rotate in the positive direction, the trend of the graph increases with the target value line.

Due to the variation in rope tension distribution caused by different height settings ($h_{1}$, $h_{2}$, $h_{3}$) during the experiments, a range of results was observed. Despite the differences in detail, a consistent trend was identified: RVCA exhibited the most effective vibration reduction, followed by input shaping and PD control.

**Figure 10:**
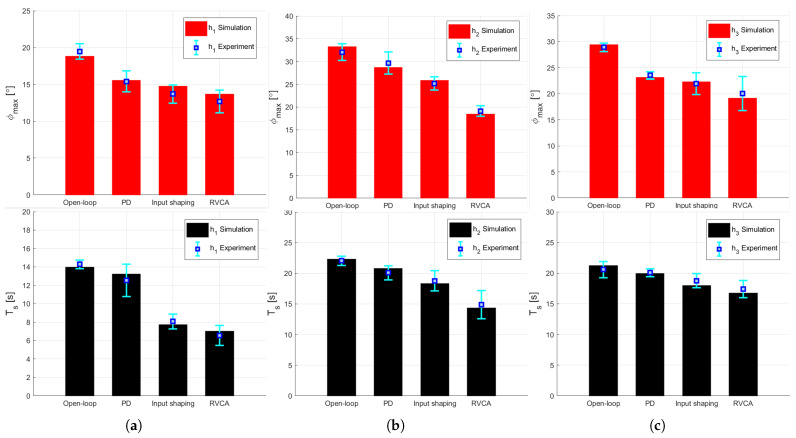
Additional experimental results are expressed in terms of the error as the degree of deviation from the simulation results. Performance comparison under four experimental conditions in terms of maximum amplitude and settling time at height (**a**) $h_{1}$, (**b**) $h_{2}$, and (**c**) $h_{3}$. Simulation values are represented by red and black bars, while experimental values and their averages are represented by blue lines and squares.

As shown in Figure 10, we conducted simulations and additional experiments to compare the maximum amplitude and settling time of the residual vibrations generated by the DAR for each experiment condition. As the simulations are based on Equation (7) and the parameter values derived from it, the reliability of the model in Equation (7) can be verified by the fact that the simulation results are within the range of the experimental results.

Table 3 depicts the average maximum amplitude and settling time of the experiments with their reduction rate compared to the open loop. Compared with the open loop results, the RVCA approach exhibited the most significant reduction in both the average maximum amplitude and the settling time, followed by input shaping and PD control, respectively. The performance of each controller was also evaluated using the root-mean-square error (RMSE) calculated from the experimental results. Compared with the open loop with an RMSE value of 6.9183°, the RMSEs of the PD control, input shaping, and RVCA were 5.1903°, 4.0391°, and 3.4135°, respectively. As shown, RVCA can be considered the best performing.

## 5. Conclusions

This study aimed to enhance the performance of the ROR system of a DAR by effectively reducing the residual vibrations. Kinematic and dynamic analyses of the ROR system yielded an accurate empirical model of the system. Using the parameters determined from this empirical model, we proposed an RVCA to reduce the residual vibration. The RVCA was a control algorithm that combined input shaping and PD control. Experiments showed that it produced the most effective results among those obtained by individually applied controls. The RMSE was used to evaluate the performance of each controller. Furthermore, a disturbance observer was integrated to address the uncertainties caused by rope deformation and slippage. This approach enhanced the overall performance of the ROR system and provided a robust foundation for future advancements in precise and reliable control systems.

A limitation of this study is the absence of a control method in which the RMA moves in coordination with the DAR during its operation, which could have enhanced the system performance. Future studies should address this gap by developing a new mechanism that enables the coordinated motion of the RMA and DAR to improve the stability and robustness of system operation.

## Figures and Tables

**Table 1:** Mathematical notations used in model analysis.

Symbol	Description
$\vec{A_{ij}}$	Vector of fixed anchor points
$\vec{B_{i}}$	Vector of magnetic encoders
$\vec{l_{ij}}$	RMA’s vector of rope
$\vec{S_{i}}$	DAR’s vector of rope
$\theta_{ij}$	Angle between the rope and the vertical reference line
ϕ	Angle representing the magnitude of residual vibration
$x_{i}$	*x* position of robots
$y_{i}$	*y* position of robots
*k*	Spring coefficient of rope
*c*	Damping coefficient of rope
*g*	Gravitational constant
$T_{ij}$	Tension of rope
$m_{i}$	Mass of robot
*I*	DAR’s moment of inertia

**Table 2:** Detailed electronic specifications of ROR system.

Item	Manufacturer	Model
BLDC Motor	Cubemars	AK60-6
Rotary Encoder	Autonics	E30S
Magnetic Encoder	SEINFLEX	D30
IMU	E2BOX	EBIMU-9DOFv5

**Table 3:** Average experiment results for each experiment conditions and the root-mean-square error (RMSE) values. The reduction rate of each conditions compared to the open loop is represented by ▾.

Experiment Condition		Open Loop	PD	Input Shaping	RVCA
	$h_{1}$	19.48	15.42 (▾20.84%)	13.69 (▾29.72%)	12.67 (▾34.9%)
Max. amplitude($\phi_{max}$) [°]	$h_{2}$	32.04	29.67 (▾7.40%)	25.19 (▾21.38%)	19.13 (▾40.29%)
	$h_{3}$	28.92	23.56 (▾18.53%)	21.93 (▾24.17%)	20.05 (▾30.67%)
	$h_{1}$	14.29	12.56 (▾12.11%)	8.09 (▾43.39%)	6.57 (▾54.02%)
Settling time($T_{s}$) [s]	$h_{2}$	22.04	20.08 (▾8.89%)	18.78 (▾14.80%)	14.91 (▾32.35%)
	$h_{3}$	20.59	20.08 (▾2.48%)	18.78 (▾8.79%)	17.41 (▾15.44%)
RMSE(ϕ) [°]		6.9183	5.1903	4.0391	3.4135

## Data Availability

The raw data supporting the conclusions of this article will be made available by the authors on request.

## Supplementary Materials

The following supporting information can be downloaded at: https://www.mdpi.com/article/10.3390/s25082463/s1, Video S1: “Rope on Rope: Reducing Residual Vibrations in Rope-Based Anchoring System and Rope-Driven Façade Operation Robot” contains concepts and experiment videos.
